# An annotated image dataset for training mosquito species recognition system on human skin

**DOI:** 10.1038/s41597-022-01541-w

**Published:** 2022-07-15

**Authors:** Song-Quan Ong, Hamdan Ahmad

**Affiliations:** 1 Institute for Tropical Biology and Conservation, Universiti Malaysia Sabah, Jalan UMS, 88400 Kota Kinabalu, Sabah Malaysia; 2 Vector Control Research Unit, School of Biological Sciences, Universiti Sains Malaysia, 11800 Penang, Malaysia

**Keywords:** Classification and taxonomy, Viral infection

## Abstract

This paper introduces a new mosquito images dataset that is suitable for training and evaluating a recognition system on mosquitoes in normal or smashed conditions. The images dataset served mainly for the development a machine learning model that can recognize the mosquito in the public community, which commonly found in the smashed/damaged form by human. Especially the images of mosquito in hashed condition, which to the best of our knowledge, a dataset that fulfilled such condition is not available. There are three mosquito species in the dataset, which are *Aedes aegypti*, *Aedes albopictus* and *Culex quinquefasciatus*, and the images were annotated until species level due to the specimen was purely bred in a WHO accredited breeding laboratory. The dataset consists of seven root files, six root files that composed of six classes (each species with either normal landing, or random damaged conditions) with a total of 1500 images, and one pre-processed file which consists of a train, test and prediction set, respectively for model construction.

## Abstract (summary)

**Table:** 

Measurement(s)	recognition system with machine/deep learning
Technology Type(s)	Camera Device
Sample Characteristic - Organism	Aedes aegypti • Aedes albopictus

## Background & Summary

Mosquito surveillance programs are probably the most important components to prevent disease outbreaks. To monitor the population of mosquitoes, field sampling and data collection of adult mosquitoes are commonly conducted, and later, the species are classified and counted in a laboratory^1,2^. However, these standard procedures to obtain mosquito number data have key constraints, such as labor, time, and cost consumption. Since humans-as-bait traps are more effective than physical traps and the public commonly encounter mosquitoes on their own^3^, the idea of engaging the public community in a mosquito surveillance program provides an excellent alternative to collect mosquito data with spatial-temporal information. To ensure that the idea of community-based mosquito surveillance is feasible, we need to support the community with the knowledge of mosquito pest recognition. The support can be achieved with a mobile application or recognition system that able to classify the mosquito at the household level.

To address these challenges, we need to define operationally of mosquitoes in the community, where two criteria need to be fulfilled: the type of mosquito species and their conditions. For the mosquito species, according to the WHO^4^, the most prevalent viral infections are dengue, chikungunya fever, Zika virus fever, yellow fever, West Nile fever, and Japanese encephalitis, and the diseases are primarily transmitted by *Aedes aegypti* (L.), *Aedes albopictus* (L.), and *Culex quinquefasciatus*. The condition of the mosquito that is likely to be observed by the community is either landing alive or smashed or damaged on human skin. Therefore, we present an annotated dataset that able to be used for training a mosquito recognition system that able to distinguish the mosquito species in harsh condition.

**Fig. 1:**
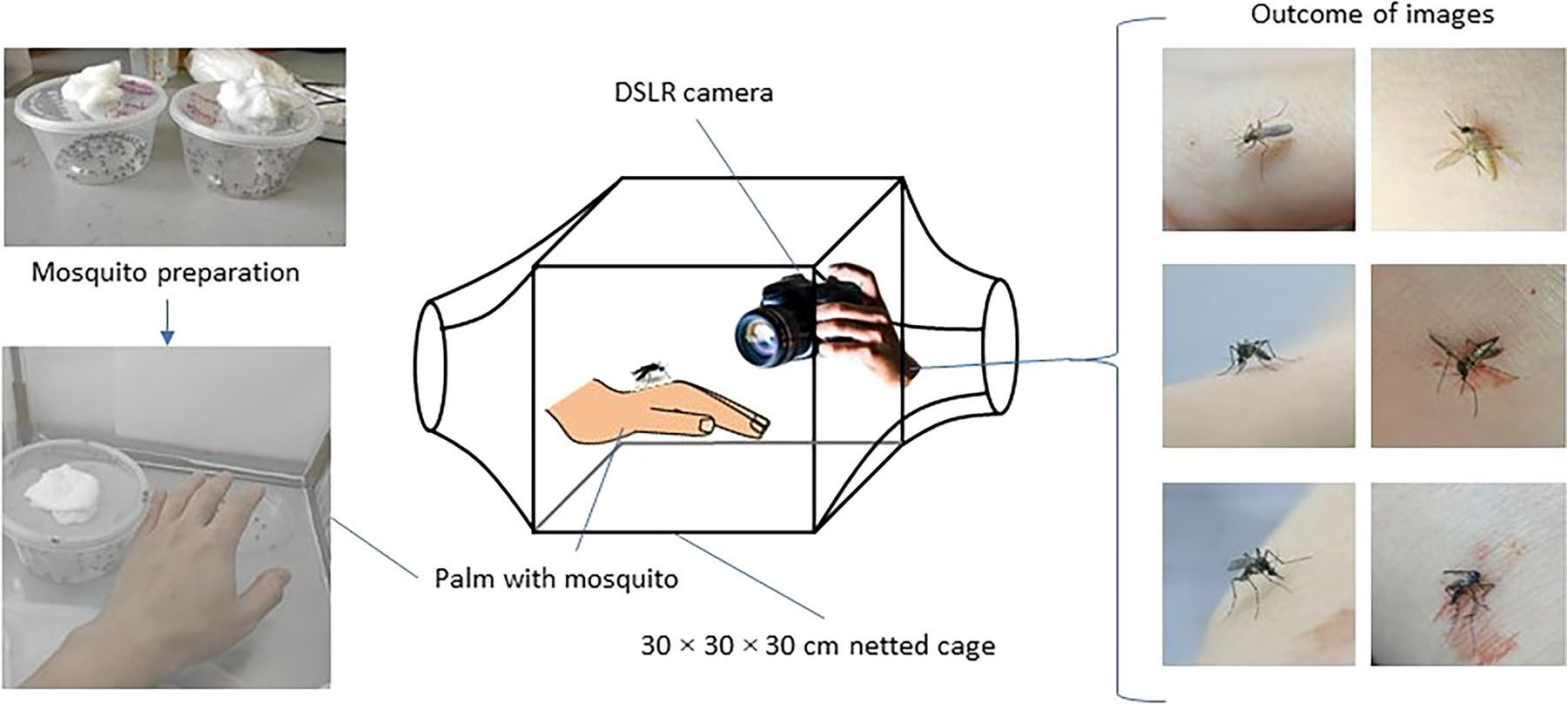
Outline of mosquito preparation and image collection.

In general, the mosquitoes were bred and grew to adult stage, 4–5 days old in a fully control laboratory, Vector Control Research Unit, Universiti Sains Malaysia, which is accredited by WHO for insecticides susceptibility test^5^. The data collection process was illustrated in Fig. 1.

**Fig. 2:**
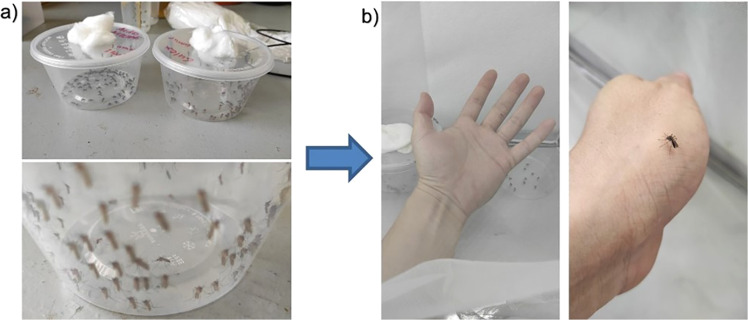
(**a**) Mosquitos’ colonies and culture from VCRU USM. Mosquito was released one by one for image acquisition, (**b**) The process Image acquisition is carried out within a 30 × 30 × 30 cm netted cage with 36 W LED Ring Light white colored illumination (5500 K).

**Fig. 3:**
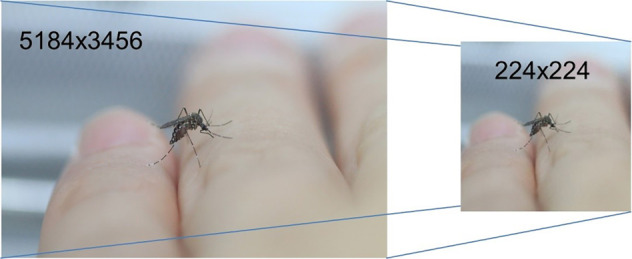
All the images were resized into 224 × 224 pixels from the original dimension.

The mosquito obtained from the mosquito breeding was transferred by a Polyethylene terephthalate (PET) container (diameter 12 cm, height 6 cm, Fig. 2a) to the net cage for image acquisition. The container and camera were placed in the cage for 30 minutes to allow the mosquito to adapt to the environment before images acquisition. The images were acquired by a digital single-lens reflex (DSLR) camera (Canon 7D, 18MP APS-C CMOS sensor, ISO 3200, auto white balance) with Tamron SP AF 90 mm f/2.8 Di Macro Lens. The images acquisition was performed on 4- to 5-day-old females’ adult in a netted cage with 34 W white light illumination on top of the cage. The volunteer consists of three ethnicities – Malay, Chinese and India, which aim to reflect the diversity of human skin tone. The volunteer’s palm is rest in the cage and different angles of the landed mosquitos’ images were acquired. Smashed mosquitoes were generated by smashing the mosquito randomly by a human palm in a non-feeding, partial, or fully repletion situation (Fig. 2b). The images were saved in JPEG format in the folders according to their classes. Images were later resized from original dimension into 224 × 224 pixels, to lower the file size of the images (as lower the computational power) that required to initiate the machine learning model training pipeline (Fig. 3), which is a common input image dimension expected by most of the deep convolutional neural network such as AlexNet^6^, ResNet and VGG-16^7^

## Methods

### Mosquito

The adult of the susceptible strain WHO/VCRU of *Ae. aegypti, Ae. albopictus* and *Cx quinquefasciatus* were obtained from the Vector Control Research Unit (VCRU), Universiti Sains Malaysia. The mosquitoes were cultured in insectarium for more than 20 years and used for the WHO insecticides susceptibility test; the colonies were maintained at 27 ± 1 °C and 75 ± 5% relative humidity in insectariums. The larvae were reared in dechlorinated water and fed with lab food (Dog biscuit: yeast: milk powder: beef liver powder at a 3:1:1:1 ratio). The pupae were transferred into a 30 × 30 × 30 cm netted cage for adult emergence. The adult mosquitoes were fed with 10% sucrose mixed with a Vitamin B complex as an energy supply. Four to five-day-old female adults were used for the images acquisition.

The dataset consists of three mosquito species - *Aedes aegypti* L., *Aedes albopictus* L., and *Culex quinquefasciatus* Say in normal landing (dorsal-ventral axis is roughly perpendicular to human skin) and smashed/damaged (lateral/dorsal of mosquito’s thorax is touch on human skin) condition, respectively. Table 1 summarized the labels, descriptions, and examples of the images in the dataset.

**Table 1:** Description, Labels, and Example of images for the dataset: Six root files that represent six classes of mosquitoes, and one pre-processed file.

Six root files of raw image data				
Sample of images	Labels	Species	Conditions on human skin	Number of images
	*Aedes aegypti* landing	*Aedes aegypti* L.	Normal landed	250
	*Aedes aegypti* smashed		Smashed or damaged	250
	*Aedes albopictus* landing	*Aedes albopictus* L.	Normal landed	250
	*Aedes albopictus* smashed		Smashed or damaged	250
	*Culex quinquefasciatus* landing	*Culex quinquefasciatus* Say	Normal landed	250
	*Culex quinquefasciatus* smashed		Smashed or damaged	250
One pre-processed data file*				
data_splitting	Train			4200
	Test			1800
	Prediction			3600

*Pre-processed the image data with augmentation and data splitting.

### Ethics statements

Ethical approval for using participants palm and mosquito imaging was obtained from the ethics commission of the Universiti Malaysia Sabah (EM1012/2021). All authors confirm that we have complied with all relevant ethical regulations.

## Data Records

The image dataset consists of six root files which are raw image data of three mosquito species with two conditions, respectively, and one data pre-processed file that could serve as an authenticated dataset in recognise three of the mosquitoes, and subsequently applied by potential user such as machine learning engineer, apps developer, data scientist, etc. The ultimate goal for the application can benefit in developing a more effective tools in recognise the mosquito species, which is crucial in mosquito surveillance. The dataset is publicly available in Mendeley Data, Identification number: 10.17632/zw4p9kj6nt.2^8^.

## Technical Validation

### Sources of mosquito and annotation validation

The source of mosquito adults is the pure bred of the susceptible strain of *Ae. aegypti, Ae. albopictus* and *Cx quinquefasciatus* from Vector Control Research Unit (VCRU), Universiti Sains Malaysia. The mosquitoes were cultured in insectarium for more than 20 years and used for the WHO insecticides susceptibility test^9,10^. Furthermore, before and after the image acquisition, the taxonomy of the mosquito were validated by two medical entomologist

### A pilot test with a basic model build-up

**Fig. 4:**
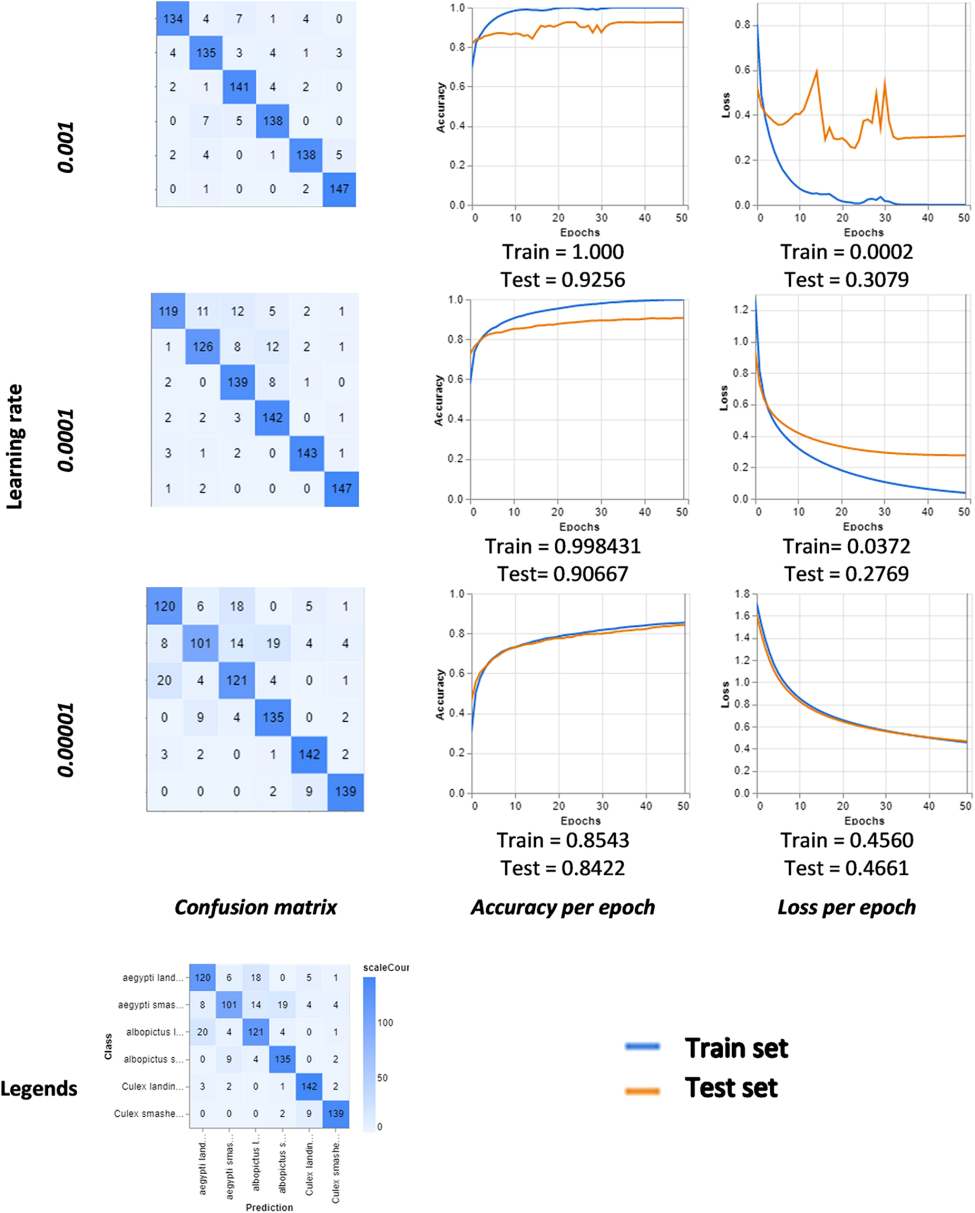
Confusion matrix, accuracy, and error loss of the pilot test of a deep learning model by using the dataset at three learning rates.

We conducted a pilot test on the datasets to validate the quality of the dataset in terms of the feasibility of deep convolutional neural networks (DCNN) model construction. We utilize a web-based tool from Google Creative Lab—Teachable Machine 2.0—that allow us to train a deep learning model with no coding required^11,12^. The data splitting and partitioning used for training and testing are: - training set (85%) and the prediction is carried out on a testing set (15%). The platform also allows us to fine-tune the model with hyperparameters, such as the learning rate, batch size, and epoch. We demonstrate the output of the models by using the datasets at three levels of learning rates - 0.01, 0.001, and 0.0001, which controls the rate of the change to the model during each step of the optimization process. Figure 4 summarises the result – confusion matrix, training, and testing accuracy and loss, respectively for validating the dataset for deep learning model construction.

## Usage Notes

The dataset contains a data pre-processed file that has data that have been augmented with four degrees of rotation – 0°, 90°, 180°, 270°, and partitioned into a training and testing set, and one prediction set to evaluate the model performance. Therefore, the file directory in Mendeley can be used directly as a URL and imported into the programming environment. Nevertheless, the dataset posted some limitations as below:

1. Lack of human skin tone diversity. The volunteers that participated in this dataset were Asian, and therefore is not covering the skin tone background of American, African, European, and Australian
2. Image data were taken in a high-resolution camera and under standardized laboratory conditions. The images were acquired by using a DSLR camera and under a condition of enough light illumination. Therefore, images from a smartphone that have been internally processed to enhance the visualization of an image and images from the field may not be recognized by the model that constructed by this dataset.
3. The dataset consists of only three mosquito species. The dataset consists of two visually similar species – *Aedes aegypti* and *Aedes albopictus*; nevertheless, other visually similar mosquitoes such as *Armigeres* are not covered in the dataset.

## Data Availability

There is no customized code in generation or processing of datasets.
